# Supplementary material for: Metabolic shift and the effect of mitochondrial respiration on the osteogenic differentiation of dental pulp stem cells
Source: PeerJ. 2023 Apr 21;11:e15164. doi: 10.7717/peerj.15164 (PMC10124543; doi:10.7717/peerj.15164)
Supplement: Supplemental Information 1 [file peerj-11-15164-s001.docx]

Supplement table 1. the sequences of specific primers and Tm value

| Gene | Primers | Tm (℃) |
| --- | --- | --- |
| *COL-1* | Forward: 5'-AAGGAGAAAGAGGAGCCAAAGG-3' | 61.1 |
|  | Reverse: 5'-AGCACCAGGGAAACCAGTCATAC-3' | 62.1 |
| *ALP* | Forward: 5'-CAGATGAAGTGGGAGTGCTTGT-3' | 59.2 |
|  | Reverse: 5'-CTGATGTGGAGTATGAGAGTGACG-3 | 59.0 |
| *TFAM* | Forward: 5'-ATGGCGTTTCTCCGAAGCAT-3' | 62.2 |
|  | Reverse: 5'-TCCGCCCTATAAGCATCTTGA-3' | 60.4 |
| *NRF1* | Forward: 5'-AGGAACACGGAGTGACCCAA-3' | 62.9 |
|  | Reverse: 5'-TATGCTCGGTGTAAGTAGCCA-3' | 60.4 |
| *GAPDH* | Forward: 5'-CTTTGGTATCGTGGAAGGACTC-3' | 58.2 |
|  | Reverse: 5'-GTAGAGGCAGGGATGATGTTCT-3' | 57.6 |
