# Supplementary figures and images for: Metabolic shift and the effect of mitochondrial respiration on the osteogenic differentiation of dental pulp stem cells
Source: PeerJ. 2023 Apr 21;11:e15164. doi: 10.7717/peerj.15164 (PMC10124543; doi:10.7717/peerj.15164)

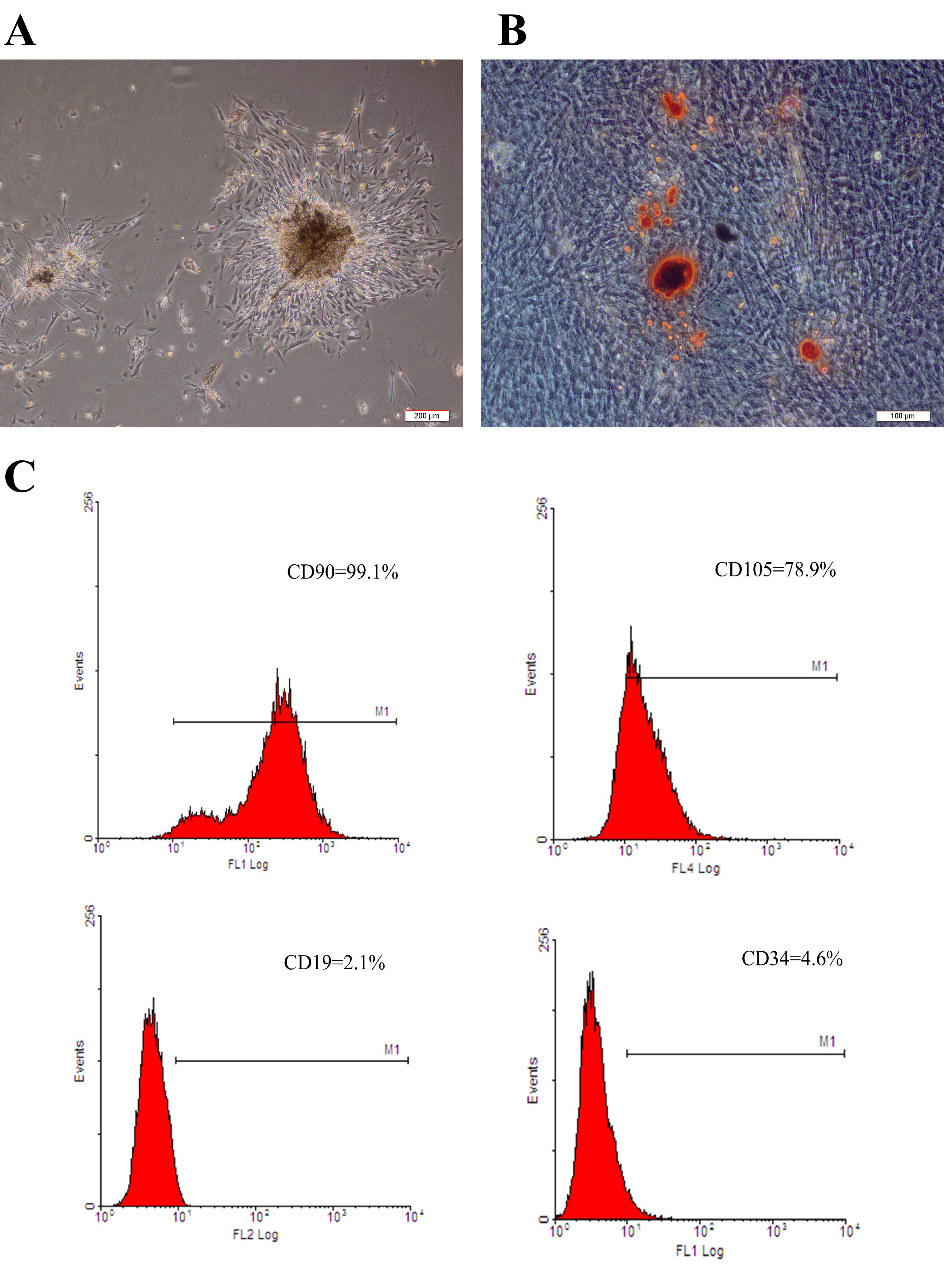

Supplement: Supplemental Information 2 — (A) phase-contrast microscopy images of hDPSCs at primary passage. (scale bar, 200 µm) (B) Alizarin red staining of hDPSCs cultured in osteogenic mineralized medium . Accumulation of mineralized nodules was observed. (Yellow arrow, scale bar, 100 µm) (C) The expressions of cell surface markers were investigated by using flow cytometry. It indicated that cells were mostly positive for CD90 and CD105 (MSCs surface markers), while they were negative for CD19 and CD34 (HSCs surface markers). [file peerj-11-15164-s002.png]
