# Supplementary material for: Metabolic shift and the effect of mitochondrial respiration on the osteogenic differentiation of dental pulp stem cells
Source: PeerJ. 2023 Apr 21;11:e15164. doi: 10.7717/peerj.15164 (PMC10124543; doi:10.7717/peerj.15164)

Some blots didn’t show markers because they were arranged in ONE membrane with only ONE protein marker

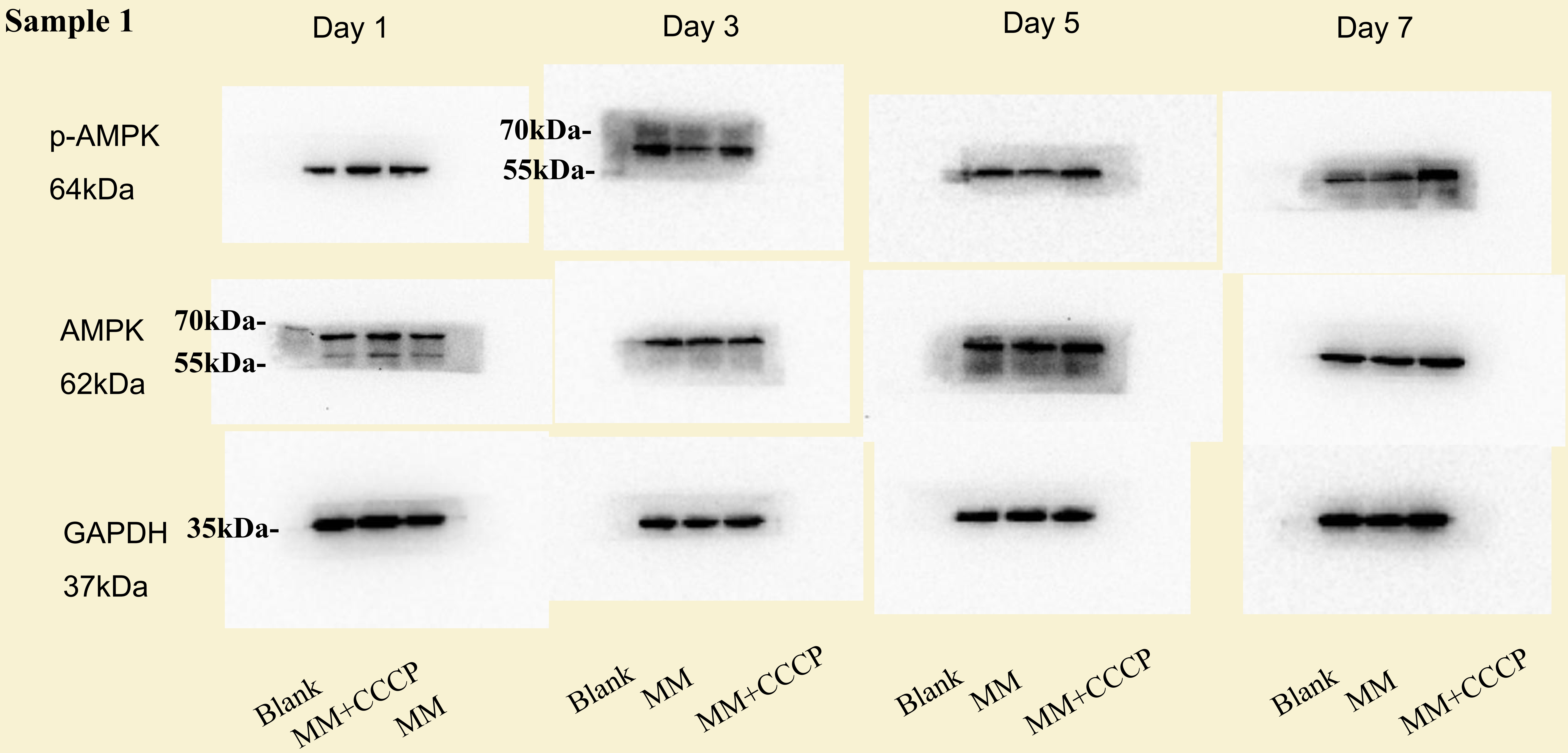

Supplement: Supplemental Information 7 [file peerj-11-15164-s007.zip › AMPK-WB-SAMPLE1.pdf]
